# Could cognitive estimation ability be a measure of cognitive reserve?

**DOI:** 10.3389/fpsyg.2015.00608

**Published:** 2015-05-07

**Authors:** Guido E. D'Aniello, Gianluca Castelnuovo, Federica Scarpina

**Affiliations:** ^1^Psychology Research Laboratory, Istituto di Ricovero e Cura a Carattere Scientifico Istituto Auxologico Italiano, Ospedale San GiuseppePiancavallo, Italy; ^2^Department of Psychology, Università Cattolica del Sacro CuoreMilan, Italy; ^3^“Rita Levi Montalcini” Department of Neuroscience, University of TurinTurin, Italy

**Keywords:** cognitive estimation, cognitive reserve, executive functions, crystallized intelligence, neuropsychological assessment

## The cognitive estimation ability: a matter of discuss

The first attempt to measure cognitive estimation abilities was made by Shallice and Evans (1978), who developed the Cognitive Estimation Test, consisting of a set of questions which requires appropriate reasoning abilities. The authors stressed the link between executive functions and estimation skills, finding out that patients with frontal lobe damages performed poorly on the proposed task; although the relation between cognitive estimation ability and executive functions is been a matter of discussion (Spreen and Strauss, 1998; Appollonio et al., 2003; Barabassy et al., 2010; D'Aniello et al., 2015), it has been recently supported by MacPherson et al. (2014), who developed a new version of the cognitive estimation test, proving its suitability for assessing executive dysfunction; in a nutshell, it seems that the process of estimation requires a complex pattern of abilities, including executive functions.

Brand et al. (2003) proposed that cognitive estimation would follow a route from a specific representation in the working memory to an activation of information from the long-term memory and- subsequently—a “plausibility check” of the generated answer by a central processing control. According to this, a cognitive estimation task would involve (i) a central processing control, which defines the appropriate strategies to solve the problem, (ii) working memory, which applies these strategies, (iii) long term declarative memory, where the necessary information for answering the task are stored.

**Figure 1:**
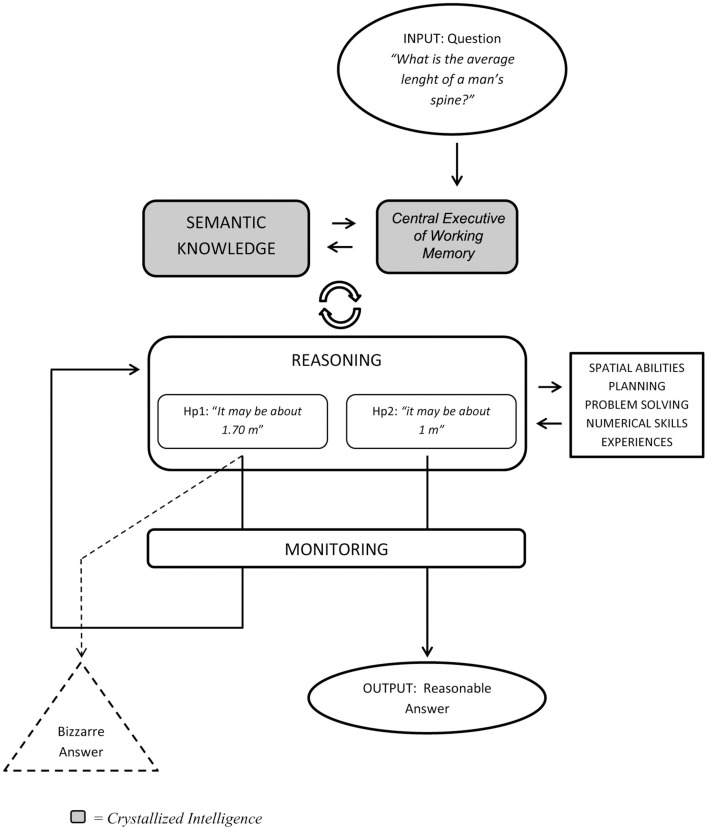
**Model of the cognitive estimation process**.

We accounted this idea in a detailed cognitive model (Figure 1) that may enclose the cognitive domains involved in the estimation process; furthermore, a more detailed characterization of the central processing control described by Brand et al. (2003) has been provided.

## A revised model

### Part I: semantic knowledge and its relation with the hypothesis of cognitive reserve

A verbal question relative to a cognitive estimation (e.g., “*what is the average length of a man's spine?*”) should firstly activate the *central executive of working memory* (Baddeley and Hitch, 1974; Baddeley, 2000), which is crucial in order to set a link between the information provided by the question and the *semantic knowledge*. The established interface between *working memory* and *semantic knowledge* is exploited by the first level of executive processing, the *reasoning abilities*: the result of this stage is the formulation of a hypothesis. Depending on the question's contents (e.g., time, quantity, size or other type of information) the *reasoning* system would recruit additional executive skills, such as *spatial abilities, planning, problem solving, numerical skills, experiences, and mental imagery* (Horacek et al., 2010). The hypothesis generated by this complex process, in which the previous cognitive modules interact with each other, is then checked by the second main level of executive processing, that is, *monitoring*: at this stage, the formulated hypothesis is compared with all the information gleaned during the process in order to verify its consistency and—once accepted—proposed as the final answer. In case the hypothesis is considered as an inconsistent or inadequate one, it is sent back to the reasoning process. This process substantially differs from reasoning abilities applied on unknown information in novel contexts (fluid intelligence, Cattell, 1967), about which, for example, the Weigl Color-Form Sorting Test (Goldstein and Scheerer, 1941) would represent an example (Hobson et al., 2007). On the contrary, cognitive estimation is strictly linked (and depends) to a certain amount of previously acquired knowledge and experience as well as the ability to use it (Della Sala et al., 2003): this combination of abilities could be defined as *crystallized intelligence* (Cattell, 1967).

The definition of crystallized intelligence as stated above is surprisingly similar to the widely known concept of Cognitive Reserve. According to Stern (2009), cognitive reserve can be defined as the amount of cognitive loss that can be sustained by brain-damaged patients before reaching a threshold at which a clear cognitive impairment becomes evident.

The estimation of cognitive reserve has been traditionally conducted using tests of crystallized intelligence, such as vocabulary, reading ability and general knowledge (Cattell, 1971; Sumowski et al., 2010). The Wechsler Adult Intelligence Scale—Information Subtest (Wechsler, 1997) has been widely used as an appropriate proxy for cognitive reserve, although it was originally intended as an instrument for assessing crystallized intelligence (Franchow et al., 2013): as confirmed by Stern (2007), measures of crystallized intelligence could provide a reliable instrument for assessing one's cognitive reserve, because they serve in capturing mature cognitive ability, even at a premorbid level (Sachdev and Valenzuela, 2009). What seems to be increasingly clear is that crystallized intelligence could represent almost part of the cognitive reserve construct, which is likely to comprise other factors including educational attainment (Stern et al., 1992; Stern, 2002; Tucker and Stern, 2011) and certain cognitive domains such as verbal fluency, reasoning, attention and working memory functions (Roldán-Tapia et al., 2012).

Given these findings, we hypothesize that assessing cognitive estimation abilities might be considered a reliable strategy for measuring important aspects of cognitive reserve: in fact, it could be used as a measure of the person's body of knowledge and experiences (Della Sala et al., 2004), independently from aging (Della Sala et al., 2004; MacPherson et al., 2014), and—at the same time—as a sensitive instrument for assessing certain cognitive domains such as working memory and semantic knowledge (D'Aniello et al., 2015). The hypothesis that semantic knowledge is part of the cognitive estimation ability would be confirmed by studies relative to patients with Alzheimer's Disease (Della Sala et al., 2004; Levinoff et al., 2006; Barabassy et al., 2007; Khodarahimi and Rasti, 2011), Mild Cognitive Impairment (Levinoff et al., 2006), and Korsakoff's syndrome (Taylor and O'Carroll, 1995; Brand et al., 2003), in which higher amount of errors in the estimation process comparing to healthy groups were reported.

### Part II: reasoning ability and the relationship with bizarre answers

The second part of the model regards *reasoning*, i.e., the ability to put together the information from other cognitive domain into the cognitive estimation, and *monitoring*, i.e., the process involves a systematic and continuous way of the reasoning process and to furnish a feedback about the results. According to our model, if the primary output from the reasoning process is not put through the monitoring stage and, for example, it is not checked with further everyday information (Shallice and Evans, 1978), but is directly provided as a result of the estimation, the final output would be a bizarre answer: it is an unreasonable and extremely inaccurate answer which was generally interpreted as a clue of pervasive impairment in cognitive estimation (Della Sala et al., 2003). Besides in previous studies relating to cognitive estimation (Appollonio et al., 2003; Khodarahimi and Rasti, 2011) less attention was generally given to bizarre answers (probably due to some difficulties in outlining an operational and actionable definition of the bizarreness index) it could be considered a remarkable clue about the executive domains involvement in the estimation ability: indeed Parkinson's disease (Appollonio et al., 2003), fronto-temporal dementia (Mendez et al., 1998), and focal frontal lesions (Shallice and Evans, 1978; Taylor and O'Carroll, 1995; MacPherson et al., 2014), but also schizophrenia (Khodarahimi and Rasti, 2011) and major depressive disorder patients (Barabassy et al., 2010) reported a poor performance in the cognitive estimation ability, and specifically an higher number of bizarre answers, since the lack of efficacy of executive functions involved in the cognitive estimation process.

## Conclusion

According to the present model, cognitive estimation is neither a pure ability nor a specific measure of executive functions, but it also require e certain amount of semantic knowledge and the ability to access to it. Given this, the Cognitive Estimation Test (Shallice and Evans, 1978 and following versions) may be considered as a useful instrument for the assessment of crystallized intelligence and of cognitive reserve to some extent. We hope this theoretical hypothesis will provide new insights prompting future research toward the understanding of the cognitive estimation process, together with its potential application as an instrument for assessing cognitive reserve.

### Conflict of interest statement

The authors declare that the research was conducted in the absence of any commercial or financial relationships that could be construed as a potential conflict of interest.
